# Dynamics of Parental School Involvement on Children’s Quality of Life—An Interactive Model

**DOI:** 10.3390/children13040561

**Published:** 2026-04-17

**Authors:** Helena Mocho, Cátia Martins, Elias Ratinho, Cristina Nunes

**Affiliations:** University Research Center in Psychology (CUIP), University of Algarve, Campus of Gambelas, 8005-139 Faro, Portugal; hsalcaparra@ualg.pt (H.M.); jeratinho@ualg.pt (E.R.); csnunes@ualg.pt (C.N.)

**Keywords:** children, interactive model, parental school involvement, parenting stress, quality of life, social support

## Abstract

Background/Objectives: Parental school involvement (PSI) is an adaptive construct that is sensitive to developmental and contextual changes and refers to the active and sustained engagement of parents in school- and home-based activities that support children’s educational experiences. Perceived social support can strengthen PSI by reducing parenting stress, while PSI may, in turn, mediate the effects of all the above factors on children’s quality of life (QoL). This study examined the direct and indirect associations among perceived social support, parenting stress, PSI, and children’s QoL, adopting an integrative framework encompassing multiple psychosocial dimensions. Methods: The sample comprised 358 Portuguese parents who completed a sociodemographic questionnaire and validated measures assessing PSI, parenting stress, social support, and children’s QoL. Results: Preliminary analyses showed that all four variables were strongly and significantly correlated. Path analysis revealed that the model showed satisfactory fit indices. Perceived social support was linked to lower parenting stress, which in turn was associated with reduced PSI. Meanwhile, greater involvement was associated with better children’s QoL, indicating an indirect pathway from stress to QoL via involvement. Parenting stress and PSI acted as critical mediating pathways between perceived social support and children’s well-being, the former as a risk factor and the latter as a protective resource. Conclusions: These findings highlight the central role of PSI in promoting children’s QoL and suggest that interventions aimed at increasing social support and reducing parenting stress may enhance parental engagement, with positive implications for family well-being.

## 1. Introduction

Parental school involvement (PSI) plays an important role in children’s development, academic achievement, and overall well-being (e.g., [1,2,3]), with recent studies suggesting the need for a more refined understanding of PSI—one that extends beyond observable school-based activities to include less visible, yet impactful, home-based expressions such as parental expectations and educational aspirations. This perspective reflects an emerging view that traditional definitions may overlook culturally embedded forms of PSI, particularly among urban and minoritized families [4].

Traditionally, PSI has been conceptualized across two domains: school-based involvement, encompassing interactions between parents and educational institutions, and home-based involvement, comprising practices that support learning outside the school context [5]. However, this binary framework has evolved into a more integrated and multidimensional construct. Building on this shift, research has moved beyond the home–school dichotomy to an integrative perspective that recognizes diverse parental behaviors, attitudes, and interactions, shaped by the child’s developmental stage, contextual factors, and the varying effects of different forms of PSI on children’s outcomes [1]. Within this perspective, PSI is now viewed as a continuum of parental behaviors ranging from minimal to highly engaged involvement [1,6]. Research has consistently shown that PSI is significantly related to children’s academic performance and school adjustment [7,8], but its impact varies depending on the specific components of PSI [8,9]. For example, PSI does not necessarily decline as children grow older; rather, its nature and effectiveness evolve in accordance with individuals’ developmental stage and educational level. Direct forms of PSI, such as shared reading, tend to be more beneficial during the early years. In contrast, for adolescents, higher parental expectations and constructive discussions about school-related topics appear to play a more pivotal role [1]. In terms of PSI in homework, its association with academic achievement tends to be weakest when it takes the form of support characterized by interference or excessive control (e.g., [1,10]); therefore, the quality and nature of engagement seem more consequential than the amount of time or resources invested [5].

Drawing on the bioecological framework of Bronfenbrenner and Morris (2006) [11] and the theory of parental role construction, PSI is increasingly viewed as adaptive and responsive to developmental shifts and contextual transitions [12,13]. Family communication quality, parental self-efficacy, an authoritarian parenting style, and parenting stress have emerged as significant predictors of children’s socioemotional well-being [14], with positive communication patterns and higher parental confidence being associated with better outcomes in this domain. In contrast, frequent use of authoritarian practices and elevated levels of parenting stress are linked to lower socioemotional indicators [14].

In addition to families’ demographic and socioeconomic characteristics, other psychological and contextual factors also impact PSI levels, including parental styles (e.g., [15]), parental competencies (e.g., self-efficacy [16]), parenting stress, and social support [17].

This study focuses on parenting stress and social support, as these factors provide a more detailed perspective on the emotional and relational dimensions of PSI, which tend to be overlooked in broader conceptual approaches.

In this context, parenting stress is conceptually distinct from general life stressors, such as financial strain or occupational pressure, as it stems specifically from the unique demands embedded in the caregiving role [18]. Unlike external stressors, parenting stress engages directly with the emotional and behavioral dynamics between parent and child. It has been shown to operate bidirectionally, both contributing to and being shaped by children’s adjustment difficulties [19]. This form of stress is further associated with diminished emotional sensitivity in parents, which may have a negative impact on children’s developmental outcomes [20,21]. Parenting stress can play a decisive role in shaping parent–child interactions and influencing children’s developmental outcomes [21]. Although direct empirical evidence on the link between parenting stress and PSI remains limited, theoretical models and existing studies suggest there is a meaningful intersection between the two constructs, given their shared grounding in parenting behavior [22]. Conceptually, PSI and parenting stress revolve around caregiving dynamics and are both shaped by parenting styles, indicating that they may be closely intertwined within broader parenting frameworks [22].

Another relevant factor is social support, which plays a pivotal role in buffering parenting stress and promoting more consistent engagement practices. Despite their relationship, social support and PSI represent distinct constructs: the latter encompasses active involvement in the child’s academic life, whereas social support refers to the emotional presence and perceived reliability of parental assistance. According to Yan et al. [23], social support may influence PSI in three ways: directly (e.g., through support from family members or close relationships), indirectly (e.g., by reducing parenting stress), and as a moderating factor (buffering the negative effects of stress on PSI).

Ultimately, high-quality PSI fosters children’s self-efficacy, emotional well-being, and academic success. However, the effectiveness of PSI should be understood through a developmental lens, where evolving child needs continually shape parental engagement [24,25]. PSI may further influence children’s quality of life (QoL), a multidimensional, subjective construct comprising physical, emotional, social, and academic well-being, shaped by factors such as parent–child relationships and perceived social support [26]. Indeed, Mocho et al. [27] found a significant relationship between parental engagement and children’s quality of life when assessing a PSI measure for children.

Under adverse conditions, PSI can function as a protective factor for children’s QoL, whereas parenting stress may exert a negative influence [2]. High levels of social support and low levels of parental stress are essential determinants of child adjustment and well-being, assuming greater relevance in families facing psychosocial risk. Evidence from a study conducted with a sample of psychosocially at-risk families from the same geographic region revealed that parenting stress levels were extremely high, significantly exceeding those observed in families from the general population [28]. Moreover, parents experiencing higher levels of stress had smaller overall social support networks, as well as fewer members in their emotional support networks. These findings highlight the need for research approaches that integrate both relational and psychological dimensions when examining the role of PSI in children’s outcomes.

Although PSI is widely acknowledged as a key contributor to children’s academic success and overall well-being, few studies adopt an interactional approach that simultaneously considers both relational (i.e., social support) and psychological (i.e., parenting stress) factors. By integrating these dimensions into a unified model, the present study aims to address critical gaps in the literature and to analyze the interactions between how these variables shape PSI patterns and affect children’s QoL.

**Figure 1:**
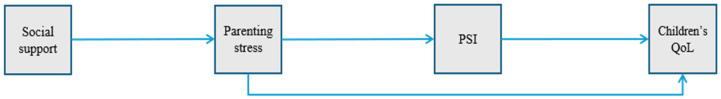
Hypothesized structural model tested via path analysis.

Therefore, this study aims to examine the direct and indirect associations between parents’ perceived social support and parenting stress with PSI, and their relationships with children’s QoL (Figure 1). Within a multidimensional framework, the study further explores how these psychosocial dimensions are related to PSI and analyzes their combined associations with children’s QoL. Consistent with these aims, the study addresses the following research question: In what ways do relational (social support) and psychological (parenting stress) dimensions contribute to PSI, and how are these contributions associated with children’s QoL?

## 2. Materials and Methods

### 2.1. Design

This study adopts a quantitative, correlational, and explanatory research design, aiming to test a theoretical interactional model of PSI. It seeks to analyze the direct and indirect effects of variables, such as perceived social support from parents and parenting stress, on PSI and on children’s QoL.

### 2.2. Participants

The sample consisted of 358 parents. From these, 83.1% were mothers, 16% were fathers, and 0.8% were guardians of children attending basic education in Portugal. Mothers aged between 27 and 56 years (*M*^age^ = 41.89, *SD*^age^ = 5.28), while fathers were between 20 and 67 years (*M*^age^ = 44.21, *SD*^age^ = 6.04). Families had between 1 and 7 children (*M* = 1.94, *SD* = 0.81), and in all cases, at least one child was between 6 and 15 years old.

Regarding the professional status of the parents, of the total number of mothers, 88.9% were employed (*f* = 312) and 11.1% were unemployed or professionally inactive (*f* = 39); with respect to fathers, 95.8% were employed (*f* = 323), and 4.2% were unemployed or professionally inactive (*f* = 14). About the academic qualifications of the mothers, 56.7% had higher education degrees (*f* = 199), 31.3% completed secondary education or an equivalent vocational course (*f* = 110), and the remaining 12% (*f* = 42) had different levels of basic education. Similarly, 31.1% of the fathers had higher education degrees (*f* = 105), 44.6% completed secondary education or an equivalent vocational course (*f* = 150), and the remaining 24.3% had different levels of basic education (*f* = 82).

Concerning parents’ perception of socioeconomic stability, 16% identified as very socioeconomically stable (*f* = 58), 83.4% as moderate (*f* = 302), and 0.6% as low (*f* = 2).

### 2.3. Measures

#### 2.3.1. Parental School Involvement

Parents’ perception of PSI was assessed using the Parental School Involvement Questionnaire—Parents’ Version (PSIQ-PV; [29]), developed from the Parental School Involvement Questionnaire—Teachers’ Version (PSIQ-TV; [30]). This instrument consists of 24 items and provides answers on a four-point Likert scale—from 1 (not at all true) to 4 (very true). This system evaluates four dimensions: (1) parental involvement in school activities and volunteering (6 items: e.g., “I give ideas for organizing activities at school [e.g., parties, sports activities, games, etc.”]); (2) parental involvement in learning activities at home (8 items: e.g., “I make a habit of checking that my child does their homework”); (3) School-family communication (6 items: e.g., “When there is a problem with my child at school, I try to inform the teacher”); (4) School activities and parents’ meetings (4 items: e.g., “I go to the parents’ meetings called by the teacher”). The higher the score, the higher the PSI level. The total scale reveals an excellent internal consistency (*α* = 0.92).

#### 2.3.2. Social Support

The adapted DUKE-UNC Functional Social Support Questionnaire ([31]; Portuguese Version; [32]) was used to assess perceived social support. This scale is composed of 13 items, distributed into three dimensions: confidant support (6 items; e.g., “I can talk to someone about my personal and family problems”), affective support (3 items; e.g., “I receive praise and recognition when I do my job well”), and instrumental support (4 items; e.g., “I can get help taking care of my children”), rated on a five-point Likert scale, from 1 (Much less than I would like) to 5 (As much as I would like). To evaluate the families’ network, there are two open questions to identify their size (number of close friends and close family they can count on). As the score increases, the level of social support also increases. In this study, the Cronbach’s alpha coefficient for internal consistency was excellent (α = 0.93).

#### 2.3.3. Parenting Stress

Parenting stress was evaluated through the Parenting Stress Index, Short Form (PSI-SF; [33] Abidin, 1995; Portuguese Version by Santos [34,35]). PSI-SF is a self-report questionnaire that assesses the parenting stress perceived by parents. It comprises 36 items, rated on a five-point Likert scale, from 1 (Strongly Disagree) to 5 (Strongly Agree), distributed into three subscales: parental distress (12 items; e.g., “I feel trapped by my responsibilities as a parent”); the parent–child dysfunctional interaction (12 items; e.g., “My child smiles at me much less than I expected”); and the degree to which the parent estimates their child as a difficult child (12 items; e.g., “My child gets upset easily over the smallest thing”). Higher scores indicate greater levels of stress in parenting. In this study, the total scale revealed excellent internal consistency (α = 0.91).

#### 2.3.4. Children’s Quality of Life

To assess parents’ perceptions of their child’s quality of life (QoL), the short form of the KIDSCREEN-52 [36,37] was used, namely the KIDSCREEN-10. This unidimensional instrument evaluates the Quality of Life (QoL; [38]). It comprises an initial item assessing the child’s overall health status (“In general, how would you describe your child’s health?”), rated on a five-point Likert scale ranging from 1 (Very poor) to 5 (Excellent), followed by 10 items that measure the child’s QoL (e.g., “Has your child felt sad?”), answered on a five-point Likert scale (1 = not at all to 5 = extremely). Lower total scores indicate feelings of unhappiness, dissatisfaction, and a sense of inadequacy across key life contexts (i.e., family, peer group, and school environment). In contrast, higher scores reflect perceived happiness, adequacy, and satisfaction with these contexts [38]. In the current sample, the internal consistency coefficient was acceptable (*α* = 0.71).

#### 2.3.5. Sociodemographic Data

A sociodemographic questionnaire was developed to gather information about parents’ sociodemographic and family characteristics. These include the following: sex, age, nationality, marital status, household composition, number of children, sex of children, children’s year of schooling, parents’ academic qualifications, parents’ profession, professional situation, and financial stability.

### 2.4. Procedure

After approval from the Data Protection Officer and the Ethics Committee of the University of Algarve, Portugal (CEUAlg Pnº 1/2024), an online questionnaire was created using the Google Forms platform. Participants were recruited through contacts with public and private schools, primary school student associations, tutoring centers, and by the snowballing sampling technique. All the questionnaires were completed in digital format, by sharing the link via email or social networks. All participants authorized their participation in the study through informed consent and were advised of the study’s aims, the anonymous and confidential nature of their responses, and the possibility of withdrawing from the study at any time without any consequences.

The answers were collected digitally in a private, controlled database between October 2024 and February 2025, with an average completion time of 30 min.

### 2.5. Data Analysis

Data were analyzed using IBM SPSS Statistics (Version 30.0; [39]). The database was screened for data entry errors; observed values were verified against allowable ranges, and no missing values were detected. Scale and factor scores were computed, and internal consistency was evaluated with Cronbach’s α (scores above 0.60 were considered unsatisfactory, while those above 0.70 were seen as adequate; [40]). Multivariate outliers were identified via Mahalanobis distance, and outlying cases were removed (*n* = 4). Descriptive statistics were used to analyze participants’ characteristics and all scales. Pearson correlation values among the four scales were estimated, with 0.20–0.39 considered as low, 0.40–0.59 as moderate, 0.60–0.79 as high, and 0.80 or above as very high. The statistical significance level was set at *p* ≤ 0.05. Path analysis was conducted in R (R version 4.5.1; lavaan version 0.6-19; [41]), and model fit was evaluated using the comparative fit index (CFI), the Tucker–Lewis index (TLI), the root mean square error of approximation (RMSEA), and the standardized root mean square residual (SRMR). According to established cutoff criteria, CFI and TLI values range from 0 to 1, with values above 0.95 indicating good model fit; the RMSEA should be below 0.08, with a 90% confidence interval whose upper bound does not exceed 0.10 and lower bound does not exceed 0.08; and for the SRMR, lower values reflect a better fit, with 0.01 considered excellent, 0.05 good, and 0.08 acceptable [42,43].

## 3. Results

To examine the strength and direction of the relationships between PSI, parenting stress, perceived social support, and perceived children’s QoL, Pearson correlation analyses were conducted (*N* = 358). The results revealed statistically significant associations among all variables (Table 1). Parenting stress showed a moderate negative correlation with perceived social support (*r* = −0.43, *p* ≤ 0.001) and a weaker negative correlation with PSI (*r* = −0.21, *p* ≤ 0.001) and with children’s QoL (*r* = −0.27, *p* ≤ 0.001). Perceived social support among parents was positively correlated with PSI (*r* = 0.21, *p* ≤ 0.001) and with children’s QoL (*r* = 0.19, *p* ≤ 0.001). A moderate positive correlation was also observed between PSI and children’s QoL (*r* = 0.32, *p* ≤ 0.001). Overall, the results suggest that higher levels of parenting stress are associated with lower perceived social support, reduced PSI, and poorer parental perceptions of the children’s QoL. In turn, higher levels of perceived social support and greater PSI are associated with improved general health and quality of life in their children.

The significant correlations observed among the variables provide theoretical support for a path-analytic model based on hypothesized causal relationships. Path analysis was conducted to assess the adequacy of a model that includes both direct and indirect effects among the constructs under investigation.

**Figure 2:**
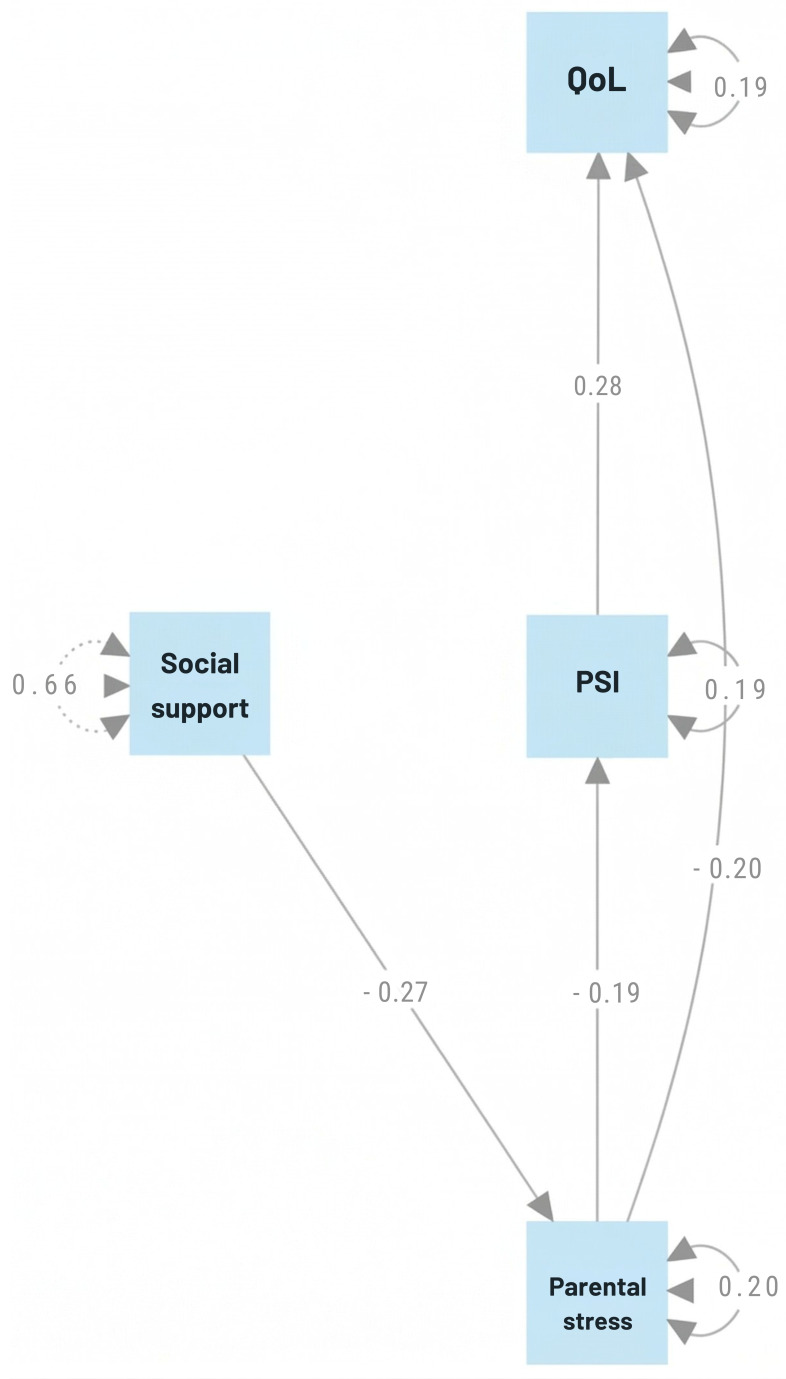
Path diagram of the structural model linking parental factors to children’s QoL.

The proposed model includes four latent constructs: perceived social support perceived by parents, parenting stress, parental involvement, and children’s QoL. Specifically, it posits that perceived social support negatively predicts parenting stress, while parental involvement acts as a mediator between support and stress. In turn, both parental involvement and perceived support are considered direct predictors of the child’s QoL. A path analysis was used to test this model and examine both direct and indirect relationships among the variables (Figure 2).

The model exhibits an overall satisfactory goodness-of-fit (Table 2). The chi-square test (*p* = 0.059; *df* = 2) did not reach statistical significance, indicating no substantial discrepancy between the observed data and the values estimated by the theoretical model. This outcome underscores that the model’s structure closely approximates the covariance patterns among variables within the bounds of the analyzed sample. Accordingly, the global fit of the model is deemed acceptable.

In terms of incremental/relative fit indices, the CFI attained a value of 0.97, indicating excellent fit, while the TLI yielded 0.90, which is generally considered acceptable. These metrics suggest that the proposed model accounts for substantially more variance than a null model, lending empirical support to its theoretical adequacy (Table 2). Regarding absolute fit indices, the RMSEA was estimated at 0.07 (90% CI: 0.008–0.136). Although the central estimate slightly exceeds the commonly accepted threshold for good fit (<0.06), the breadth of the interval (particularly the very low lower bound) may be interpreted as indicative of a reasonable approximation, especially in light of the model’s complexity and reduced degrees of freedom. The SRMR presented a value of 0.036, confirming strong correspondence between the observed and estimated data points. Taken together, these indicators provide consistent empirical and theoretical support for the structural validity of the model (Table 2).

Based on the estimated model (Figure 1), a direct effect of social support on parenting stress was observed (*β* = −0.27, *p* ≤ 0.001), indicating that higher levels of social support are associated with lower levels of parenting stress. A similarly negative direct relationship was identified between parenting stress and PSI (*β* = −0.19, *p* ≤ 0.001), suggesting that increased parenting stress corresponds to reduced PSI. PSI and child QoL were directly and positively associated (*β* = 0.28, *p* ≤ 0.001), consistent with the premise that greater PSI contributes to improved child QoL. No direct link was observed between parenting stress and child QoL. Thus, the relationship between parenting stress and children’s QoL appears to be predominantly indirect, mediated by PSI. Thus, both parenting stress and PSI act as critical mediating pathways between social support and child well-being, albeit in opposite directions: the former representing a risk factor (stress) and the latter functioning as a protective resource (PSI).

These findings imply that increases in parenting stress diminish PSI levels, which, in turn, negatively affect child QoL, resulting in a modest overall effect that nonetheless aligns with the theoretical framework (Figure 2). Residual factors reflect variance not accounted for by observed predictors and differences in measurement scales. Accordingly, interpretation of temporal dynamics cannot be inferred, and residual factors were not considered due to the cross-sectional nature of the data. Internal paths were estimated as contemporaneous autoregressive effects, representing residual variation specific to each variable after controlling for structural effects from the included predictors. The internal path of social support (*β* = 0.66, *p* ≤ 0.001) reflects a strong contemporaneous self-dependence. In contrast, the negative coefficient observed for PSI (*β* = −0.20, *p* ≤ 0.001) may indicate a statistical pattern of compensation or regression toward the mean within the modeled interactions.

## 4. Discussion

This study aimed to examine the direct and indirect associations with social support perceived by parents and parenting stress with PSI, and their relationships with children’s QoL. Within a multidimensional framework, it explores how these psychosocial dimensions are related to PSI and analyzes their combined associations with children’s QoL.

The results found support PSI as an important contributor to child QoL and reveal relational patterns among parental psychosocial variables, helping to clarify how stress and perceived social support may be associated with family dynamics and parental perceptions of their children’s QoL. The findings show significant associations among parenting stress, perceived social support, PSI, and parental perceptions of children’s QoL. Thus, parenting emerges from a dynamic interplay among child traits, parental characteristics, and broader social influences, reflecting a complex and interrelated set of determinants [44]. In line with the findings of Mocho et al. [2,3], our results reinforce the notion that PSI is a significant predictor of children’s QoL. This association appears to be strengthened by the psychosocial and socioeconomic stability of the family environment, highlighting the effects of contextual determinants in shaping children’s overall well-being during early developmental stages [2]. They also corroborate previous research in which parenting stress emerged as a robust predictor of children’s socioemotional and academic well-being, with lower stress levels among parents being associated with better outcomes in both domains [14]. This relationship was reflected in our data, with lower parental stress associated with more positive outcomes in both areas.

The moderate negative correlation between parenting stress and perceived social support underscores the protective role of supportive environments in mitigating caregiver stress levels. In this context, affective social support constitutes an important protective factor for child adjustment, as lowering it may be associated with higher levels of parenting stress, resulting from diminished support in children’s education and reduced opportunities to cope with the demands of parenting [45]. Furthermore, the impact of social support on parenting may depend not only on its presence but also on its quality, origin, and relevance; however, the scarcity of longitudinal research, particularly among typical populations, limits the ability to distinguish between general and protective effects. Despite mixed outcomes, the evidence underscores the significance of context and shows that social support influences parents in diverse ways [44].

Additionally, the negative associations observed among parenting stress, PSI, and child QoL suggest that elevated stress may hinder parental participation and collaboration in practices that support their children’s academic journey, as well as affect their subjective evaluation of child well-being. According to Jones et al. [46], high levels of parenting stress may thwart constructive interactions with children, potentially intensifying family discord, and this escalation can exacerbate children’s difficulties and sustain a recurring pattern of stress and conflict. Parents experiencing higher levels of stress tend to be less emotionally and physically available, which undermines the quality of parent–child interactions, leading to increased stress in the child [46] and, inevitably, a lower perceived QoL. Empirical evidence suggests that PSI may serve as a mediator in the relationship between psychological stress and QoL [47]; conversely, however, more robust perceived social support may facilitate more active PSI.

The positive association between PSI and children’s QoL confirms the predictive relevance of the former, highlighting the importance of interventions aimed at strengthening this variable to mitigate the impact of parenting stress. This relationship corroborates early findings [2,3], further highlighting the fact that, during periods of adversity, PSI emerges as a significant explanatory factor for children’s QoL, underscoring its protective role in fostering well-being under challenging circumstances.

In line with the evidence reported by Cheng et al. [47], psychological stress appears to affect family QoL through PSI, which may operate as a full or partial mediator. Despite the robustness of these observed effects, the cross-sectional nature of the study limits causal inferences; therefore, interpretations of internal paths and compensation patterns should be approached with caution, as the data do not support causal or longitudinal conclusions. Nevertheless, cross-sectional designs present substantial advantages that support their adoption and highlight their value in research. They are often praised for their simplicity, cost-effectiveness, short data-collection periods, and reduced participant burden, all of which make them especially suitable for capturing a clear, time-specific snapshot of the phenomena under investigation [48]. Given this study’s use of Pearson correlations and path analysis, such a design is well-suited to examining theoretically grounded relationships among variables and to mapping patterns of association and compensation, while refraining from making any direct causal claims [48].

These findings may offer relevant contributions to practitioners, families, and researchers by providing evidence that reducing parenting stress and strengthening PSI can operate as complementary mechanisms to improve children’s QoL. Grounded in the observed associations, this study supports the development of targeted interventions that integrate these dimensions to foster supportive family environments and promote positive child development.

### Limitations and Suggestions for Future Research

This study has some limitations that should be considered when interpreting the findings. Most notably, the model was estimated using cross-sectional data, which restricts conclusions regarding the directionality or causality of the relationships among variables. The absence of longitudinal measures further limits the ability to capture dynamic processes inherent in the model. As such, caution is advised when interpreting internal paths and autoregressive effects, which reflect local residual dependence rather than temporal stability. Their inclusion served to support the statistical structure and ensure appropriate model identification.

Additionally, the model did not account for contextual variables that may influence or moderate the relationships observed. Factors such as socioeconomic status, family configuration, informal support networks, and parental mental health history could introduce meaningful variation in the associations among social support, parenting stress, PSI, and children’s QoL.

A further aspect to consider is the absence of detailed information on the children’s ages. This decision resulted from an ethical and methodological consideration, as collecting additional variables would have required a level of detail that did not align with the original study design and could have increased the sensitivity of the data requested from families. Moreover, the psychosocial variables examined in this study, namely perceived social support and parenting stress, tend to show relative stability over time, which mitigates, to some extent, the impact of age variability on the core associations explored. Although PSI may reconfigure as children progress through different developmental and educational stages, it does not necessarily diminish; rather, it tends to adjust to the child’s needs and the demands of each school level. Nonetheless, the absence of precise age distribution limits the possibility of examining developmental differences in PSI, parenting stress, and children’s QoL. Future research should therefore include children’s age as a central variable, enabling more refined analyses and a more comprehensive understanding of how these processes unfold across childhood and adolescence.

To build upon current findings, future research should consider longitudinal designs that explore temporal and causal dynamics among these constructs. It may also be relevant to examine additional mediating and moderating mechanisms, such as coparenting quality, parental resilience, and perceptions of child temperament, as these may shape or condition the pathways identified. In addition, future studies should integrate culturally grounded forms of PSI, as the recent literature suggests that traditional conceptualizations may fail to capture PSI practices that are embedded in specific cultural contexts, particularly among urban and minority families. This would strengthen the ecological validity and cultural responsiveness of PSI research.

Incorporating mixed-method approaches, including qualitative data from interviews or focus groups, could help contextualize statistical findings and reveal subjective experiences related to PSI, social support, and parenting stress. Moreover, analyzing outcomes across diverse population subgroups, such as families of children with special educational needs, those facing socioeconomic adversity, and those residing in rural versus urban contexts, could highlight how these processes operate across distinct social realities.

Due to the specificity of the target population (i.e., parents of children aged six to fifteen years), the sampling process faced logistical constraints. A nonprobabilistic snowball sampling strategy was adopted to ensure access to eligible participants, while acknowledging that this approach may limit the representativeness of the findings. Future studies may benefit from broader recruitment strategies to enhance sample representativeness and generalizability.

Despite this study’s contribution, the existing literature on this topic remains insufficient, particularly regarding typically developing populations. Accordingly, future research is encouraged to further examine the relationship between PSI and children’s QoL in normative samples, thereby enriching the breadth and representativeness of the evidence base.

## 5. Conclusions

This study offers data that help describe parental behavior patterns linked to the adjustment of Portuguese children and adolescents, underscoring the central role of PSI in promoting family well-being and children’s quality of life. All correlations among the model constructs were highly significant, reinforcing the internal consistency of the findings, and the model demonstrated a good overall fit, supporting the robustness of the observed pattern.

Parenting stress emerged as a particularly relevant variable, suggesting that its impact on child well-being occurs mainly through changes in parenting practices and PSI. When combined with social support, lower levels of parenting stress appear to foster more responsive and effective engagement, promoting substantial gains in children’s QoL. This pattern is consistent with existing evidence linking sensitive parenting practices to improved indicators of child adjustment.

Despite these promising findings, caution is warranted in interpreting the results, given the study’s cross-sectional design and the absence of contextual variables. Nonetheless, the data reinforce the importance of designing interventions that integrate parental involvement and social support as foundational pillars for the healthy development of children and adolescents, offering tangible benefits for both caregivers and children.

## Figures and Tables

**Table 1:** Pearson’s correlations of parenting stress, social support, PSI, and children’s QoL.

Dimensions	*M* (*SD*)	1	2	3	4
1. Parenting stress	1.96 (0.50)	1			
2. Social support	3.86 (0.81)	−0.43 **	1		
3. PSI	3.09 (0.46)	−0.21 **	0.21 **	1	
4. QoL	3.52 (0.47)	−0.27 **	0.19 **	0.32 **	1

Notes. *M* = Mean; *SD* = Standard deviation; PSI = Parental school involvement; QoL = Children’s health-related quality of life; ** = *p* < 0.001.

**Table 2:** Fit indices for the estimated path model.

χ^2^	*df*	CFI	TLI	RMSEA (90% CI Lower–Upper)	SRMR
*p* 0.059	2	0.967	0.901	0.071 (0.008–0.136)	0.036

Notes. N = 358; χ^2^ = Chi-Square; df = degrees of freedom; CFI = Comparative Fit Index; TLI = Tucker–Lewis Index; RMSEA = Root Mean Square Error of Approximation; SRMR = Standardized Root Mean Square Residual.

## Data Availability

The data can be made available for consultation upon request to the corresponding author due to privacy restrictions.

## Abbreviations

The following abbreviations are used in this manuscript:

PSI	Parental school involvement
QoL	Quality of life
